# State of Art of Cancer Pharmacogenomics in Latin American Populations

**DOI:** 10.3390/ijms18060639

**Published:** 2017-05-23

**Authors:** Andrés López-Cortés, Santiago Guerrero, María Ana Redal, Angel Tito Alvarado, Luis Abel Quiñones

**Affiliations:** 1 Centro de Investigación Genética y Genómica, Facultad de Ciencias de la Salud Eugenio Espejo, Universidad Tecnológica Equinoccial, Quito 170527, Ecuador; aalc84@gmail.com; 2 Gene Regulation, Stem Cells and Cancer Programme, Centre for Genomic Regulation (CRG), The Barcelona Institute for Science and Technology, Universitat Pompeu Fabra (UPF), Barcelona 08003, Spain; santiago.guerrero@crg.eu; 3 Instituto de Fisiopatología y Bioquímica Clínica, Facultad de Farmacia y Bioquímica, Universidad de Buenos Aires, Centro de Diagnóstico Molecular, MEDgenomica, Buenos Aires 1000-1499, Argentina; marianared@hotmail.com; 4 Unidad de Bioequivalencia y Medicina Personalizada, Facultad de Medicina, Universidad de San Martín de Porres, Lima 12, Peru; eaa.alvarado@hotmail.com; 5 Laboratory of Chemical Carcinogenesis and Pharmacogenetics, Department of Basic-Clinical Oncology, Faculty of Medicine, University of Chile, Santiago 70111, Chile

**Keywords:** cancer, single nucleotide polymorphism, precision medicine, Latin America, pharmacogenetics, pharmacogenomics

## Abstract

Over the past decades, several studies have shown that tumor-related somatic and germline alterations predicts tumor prognosis, drug response and toxicity. Latin American populations present a vast geno-phenotypic diversity due to the great interethnic and interracial mixing. This genetic flow leads to the appearance of complex characteristics that allow individuals to adapt to endemic environments, such as high altitude or extreme tropical weather. These genetic changes, most of them subtle and unexplored, could establish a mutational profile to develop new pharmacogenomic therapies specific for Latin American populations. In this review, we present the current status of research on somatic and germline alterations in Latin America compared to those found in Caucasian and Asian populations.

## 1. Introduction

The global increase in the cancer burden is raising an unprecedented awareness among governments of the need for action on cancer control. Countries in economic transition will experience marked increases in the number of cancer cases in the coming years as a result of rapid growth and ageing of their populations, coupled with increasing cancer rates as lifestyle and environments continue to evolve. Latin America is not an exception, with a suite of major social and demographic changes that underlie the predicted 1.7 million new cancer cases and one million cancer deaths in 2030, a 67% rise relative to 2012 estimates [1]. The incidence and mortality rates make cancer a public health problem; and strong economic investment has been made for the development of drugs sold at high prices. However, the progress on genome research in somatic and germline mutations is generating new pharmacogenomics treatments that can act in a personal or in a population manner, in accordance with the genomic profile of individuals, allowing governments and health entities to save economic resources.

Somatic and germline mutations may have a great impact on disease prognosis and/or response to therapy. Somatic mutations appear after an oncogenic insult within the tumoral tissue while germline mutations are heritable alterations found within the individual. These mutations could be used as prognostic indicators of cancer outcome and/or predictive biomarkers to optimize therapy decisions among subpopulations of patients. Thus, genetic information can be used for both the selection of effective therapy and the avoidance of treatments with an unacceptable risk of adverse drug reactions [2]. In this review, we will present current status of research on somatic and germline alterations in Latin American compared with Caucasian and Asian populations, the burden of cancer and the imperative to perform comprehensive cancer pharmacogenomics studies, which may lead to the discovery of new clinical biomarkers, driver oncogenes and/or therapeutic targets.

## 2. Somatic Genomics in Oncology

A normal cell becomes cancerous by losing its ability to properly regulate key molecular processes involved in cellular replication. This initial genetic alteration can be a simple mutation in the DNA or a deep genetic deletion or amplification. This malignant cell, which carries the founder somatic genome, proliferates and additional genetic and/or epigenetic alterations (e.g., DNA methylation) can be acquired [3]. These genomic aberrations can further provide cellular advantages for angiogenesis and metastasis progression [4]. The initial and acquired alterations determine the aggressiveness of the tumor and the sensitivity or resistance to therapy. Thus, identifying driver somatic alterations is essential to predict tumor prognosis and response to treatment [5].

Cancer research has evolved in parallel with high-throughput omics technologies, leading to the development of a personalized genomic-based therapy. This tailored treatment not only takes into account the clinical aspects of each patient, but also, and most importantly, the molecular characteristics of their tumors. Thus, to offer a precise anticancer therapy, personalized oncology identifies druggable cancer driver proteins based on their genomic alterations (somatic mutations, copy number alterations and mRNA expression) and differences between human populations [6,7].

Nowadays, for instance, anti-cancer therapy in non-small-cell lung cancer (NSCLC) is tailored according to the patient genomic signature. In NSCLC, two major driver oncogenes are found: epidermal growth factor receptor (*EGFR*) and anaplastic lymphoma kinase (*ALK*) [8]. Since *EGFR* and *ALK* mutations are mutually exclusive, *EGFR*-mutated patients are treated with EGFR tyrosine kinase inhibitors (TKIs), such as gefitinib or erlotinib, while ALK-altered patients are treated with specific ALK inhibitors, such as crizotinib or ceritinib. Concerning human populations, about 15% of Caucasian and 40–50% of Asian patients with lung adenocarcinoma present alterations in the EGFR gene. On the contrary, *KRAS* (Kirsten rat sarcoma viral oncogene homolog) mutations are observed in ~15% of NSCLC Asians patients and ~30% of NSCLC Caucasians patients. However, these alterations are found in parallel with many others and therefore *KRAS* is not considered as a driver oncogene. Other mutations have also been reported in *ROS1*, *BRAF*, *MET*, *RET*, and *Her2* genes with an incidence rate of 1–3%. Regarding *ALK* genomic subtype, 3–7% of NSCLC patients present an activated form of *ALK*, formed by a gene fusion event between *ALK* and the echinoderm microtubule-associated protein-like 4 (*EML4*). This abnormal protein is involved in cell proliferation as well as apoptosis inhibition [8].

In Chinese, Korean, and Japanese female non-smokers with lung adenocarcinoma (AD), there is a high prevalence of *EGFR* mutations clustered between exons 18 and 21. These mutations are useful biomarkers of *EGFR* TKIs response and can predict a better clinical outcome after gefitinib treatment. Thus, clinical decisions can be taken not only based on the genomic subtype but also on ethnicity [6,9].

Recently, Chen et al. (2016) analyzed The Cancer Genome Atlas Network (TCGA) NSCLC genomic database (*n* = 1023) to classify NSCLC into nine subtypes: three within squamous cell carcinoma (SQCC) and six within AD [10]. SQCC subtypes were related with transcriptional targets of *SOX2* or *p63*. Regarding the six AD subtypes, one shared molecular characteristics with neuroendocrine tumors, two showed a CpG island methylator phenotype, and three manifested high *p38* and *mTOR* pathway activation. Although detection of *ALK* and *EGFR* mutations is widely accepted as a standard procedure for NSCLC personalized therapy, this molecular classification could provide new information to detect clinical biomarkers or therapeutic targets [10].

In colorectal cancer (CRC), 5-fluorouracil (5-FU) based chemotherapy has been accepted as the first line therapy and is applied for neoadjuvant and adjuvant treatment of CRC patients. Recently, the incorporation of anti-EGFR monoclonal antibodies into the traditional chemotherapy has greatly improved the efficacy and is now also used to treat metastatic colorectal cancer (mCRC) [11,12]. However, EGFR-based therapies are only efficient in a subgroup of patients. This efficacy has been related to the mutational profile of the *KRAS* gene [13]. Several studies have clearly demonstrated that only mCRC patients with wild type (WT) *KRAS* respond to anti-EGFR treatment. *KRAS* is a member of the *RAS* gene family (*HRAS*, *KRAS*, and *NRAS*), which encodes highly similar membrane-localized G proteins [14]. Members of this family have been associated with tumorigenesis due to their implication in key cellular processes, such as cell growth and apoptosis [15,16]. In particular, *KRAS* has been implicated in several types of malignant tumors, including colon, lung and pancreatic cancer [17,18]. Several activating mutations in the *KRAS* gene, resulting in EGFR-independent activation of the mitogen-activated protein kinase pathway (*MAPK*), have been reported. The most frequent alterations are found in codon 12 (~77% of all reported *KRAS* mutations) and codon 13 (~23%) [19]. Although alterations in other positions, such as codon 61, have also been reported, they only account for 1–4% of *KRAS* reported variants. The clinical relevance of those variants in CRC and mCRC treatment is still unknown. The prevalence of *KRAS* mutations differs amongst human tumors. Previous studies have shown that the frequency of mutation is around 30–40% in CRC and we reported 32% of mutations [20]. These observations are similar when comparing different ethnic groups [18,21,22]. For instance, the mutation frequency of *KRAS G35A* (codon 12) was 0.71 and of *KRAS G38A* (codon 13) was 0.29 in the Mexican population [18]. Identifying the mutational profile of *KRAS* in each patient is required to apply the best possible treatment: patients with the wild type *KRAS* could receive monoclonal antibodies against *EGFR*, while *KRAS* mutated patients have been related with poor prognosis and no-response to anti-EGFR therapies [23,24,25].

In cutaneous melanoma, Akbani et al. (2015), proposed a classification into four subtypes based on the most significantly mutated genes: *BRAF* (47%), *RAS* (29%), *NF1* (9%), and Triple-WT (15%). These proteins are involved in the mitogen-activated protein (MAP) kinase pathway. In this process, activated RAS proteins stimulate the RAF kinases ARAF, BRAF and RAF1, which, in turn, phosphorylate MEK kinases. Activated MEK kinases phosphorylate ERK kinases that subsequently regulate multiple cellular processes involved in oncogenesis. In contrast, NF1 protein acts as a negative regulator of RAS signaling. These genomic signatures allow clinicians to administrate an accurate treatment for each patient. For instance, *BRAF*-mutated patients receive BRAF inhibitors, such as vemurafenib or dabrafenib; while *RAS* patients are likely to respond to MEK inhibitors [26].

Despite the effort in understanding the above TCGA genomic classifications, these studies have been performed using tumor samples predominantly from Caucasians. Regarding the TCGA melanoma classification, 96% of samples were obtained from White populations, leaving other populations highly under-represented: 1.4% Hispanic/Latino, 0.3% Black/African American and 2% Asian [26]. Concerning the TCGA NSCLC genomic classification, 69% of samples came from White populations, 1% from Hispanic/Latino and 30% unknown [10]. On the other hand, the PanCancer Analysis of Whole Genomes (PCAWG) Project is coordinated by the TCGA and the International Cancer Genome Consortium (ICGC), and has so far carried out 74 projects about all types of cancer in more than 25,000 genomes that mainly belong to Caucasian and Asian populations [27,28]. Nevertheless, Latin America populations have different geno-phenotypic characteristics due to the great inter-ethnic mixing and environmental aspects. For instance, Latin America populations are highly exposed to ultraviolet (UV) radiation due to high altitude cities (i.e., Quito or La Paz), tropical weather and ozone depletion in some regions (South America). In melanoma, this could greatly increase UV-based mutations and completely change the mutational landscape defined Akbani et al. (2015) [26].

## 3. Germline Pharmacogenomics in Oncology

For more than 50 years, it has been proven that the genetic differences among people contribute to interindividual responses to drugs commonly used in cancer treatments. Pharmacogenetics and pharmacogenomics determine these genetic differences to predict drug safety, toxicity and efficiency among individuals and populations. Pharmacogenetics studies the variability in drug response due to heredity focusing on genes implicated in drug metabolism, while pharmacogenomics enlarges the understanding of drug response by studying all genes in the genome. The term pharmacogenomics was associated with the Human Genome Project (HGP). According to the HGP, 99.9 percent of the information found in around 23,000 human genes is identical among individuals, whereas the remaining 0.1 percent of genes is specific to each individual. This difference may be influential in the susceptibility to develop any type of cancer or to determine how an individual reacts to different treatments and how drugs are metabolized [29,30].

The term pharmacogenetics encompasses two disciplines: pharmacology and genetics. Pharmacology aims to understand how drugs act on the body and genetics is the study of genes and their variation and heredity among individuals. Thus, pharmacogenetics tries to determine the genetic cause of unexpected drug responses [31]. It focuses on the role played by the genetic variation in pharmacokinetics, such as drug absorption, distribution, metabolism, and excretion; as well as in pharmacodynamics, such as drug-response proteins, receptors, channels, and transporters [29,30,32]. On the other hand, pharmacogenomics determine genetic differences in a population-based manner that may explain drug response and toxicity. The human genome is composed of 3.1 billion nucleotide bases and genetic variations can be found among human populations. These variations can be classified in single nucleotide polymorphisms (SNPs), deletions, amplifications, insertions or tandem repeats [33]. These variants, which are overall considered as the germline genome, can be present in genes that codify for proteins implicated in key molecular processes that affect drug response [5]. In this section, we describe polymorphisms in drug transporters, drug targets, and drug-metabolizing enzymes.

### 3.1. Single Nucleotide Polymorphisms

During the 1990s, after digesting DNA sequences, the existence of variations in the cutting sites that exactly belonged to a single nucleotide was found. Today, the 1000 Genomes Project Consortium (phase 3) identified about 84.7 million SNPs in 26 human populations, along with 3.6 million short insertions/deletions (indels) and 60,000 structural variants [34]. Likewise, the variants can be found both in coding sequences called exons and non-coding sequences called introns, and can be responsible for a higher or lower tendency to develop illnesses. The SNPs may or may not cause changes in the essential information of nucleotides, and, therefore, the gene, but regardless of the case, their relation to pathologies, such as different types of cancer, is becoming clearer [35].

The SNPs in drug-metabolizing enzymes, transporters, and receptors have relevant effects in the efficiency and toxicity of some drugs [36,37]. Consequently, science and technology make progress everyday in order to create drugs that can be used in personalized treatments for patients suffering from different types of cancer. Despite the fact that environmental factors, age, type of nutrition, and health condition may be influential in the response to the pharmacological treatment, the genomic information is the key to creating personalized drugs with greater efficacy and safety. The SNPs are the most common variation in the DNA sequence, whereas mutations are uncommon variations, but the main cause of genetic disorders [38]. From the point of view of evolution, SNPs are interesting to analyze. First, the frequency of SNPs in exons and introns is very similar: 1/346 base pairs (bp) and 1/354 bp, respectively. The SNPs of exons could be related to illnesses, whereas the SNPs of introns, especially all the perigenetics, are related to variations in the alternative splicing and non-expression sites for miRNAs. When comparing SNPs between chimpanzees and humans, little variations in number (0.6%) have been found. After analyzing their distribution in the genome, the difference is of some 32%. Additionally, it has been discovered that the SNPs vary among populations. For instance, African American people and African people have more SNPs than Europeans and Asians (93:17) [34].

### 3.2. Drug Transporters

Pharmacogenomics identifies the inherited genetic variations that may predict patients’ response to different types of chemotherapy more efficiently. The genetic variations significantly change among ethnic groups, and the assessment of the haplotypes may generate results that are highly correlated to the phenotype [39].

Considering the polymorphisms in drug transporters, the *SLCO1B1* gene presents the SNP SNP rs4149056, also known as T37041C, in the exon 5, chromosome 12. This gene encodes the organic anion-transporting polypeptide 1B1 (OATP1B1) protein, which is a membrane transport protein located mainly in the liver and regulates the circulation of organic anions [40,41]. Several SNPs (i.e., T37041C) that are involved in the folate pathway have been implicated in the pharmanokinetics and effects of methotrexate [42]. In addition, this polymorphism increases systemic exposure to simvastatin and the risk of muscular toxicity [43,44,45]. According to the 1000 Genomes Project (phase 3), the allele frequency of T37041C is 0.82 (T) and 0.18 (C) in Colombia, 0.92 (T) and 0.08 (C) in Mexico, 0.86 (T) and 0.14 (C) in Peru, and 0.88 (T) and 0.12 (C) in Puerto Rico, in contrast with 0.86 (T) and 0.14 (C) in Han Chinese population, 0.86 (T) and 0.14 (C) in British population, and 0.88 (T) and 0.12 (C) in Iberian population [34] (Table 1).

### 3.3. Drug Targets

As for the polymorphisms in drug targets, 5-fluorouracil (5-FU) inhibits the thymidylate synthase (*TYMS*) enzyme through dUMP. The thymidine triphosphate is essential to repair and synthesize the DNA. It is generated from the *TYMS* [46]. The *TYMS* inhibition is a well-known target for 5-FU. However, there is evidence regarding the overexpression of *TYMS* in tumors and the resistance to the TYMS-targeted agents [47]. The *TYMS* expression levels are regulated by polymorphic tandem repeats in the *TYMS* enhancer region (TSER) (rs34743033); the more repeats, the more enzyme activity. Therefore, three tandem repeats (TSER*3) have higher mRNA expression levels in the tumor tissue unlike TSER*2 and that is correlated to a lower response reported to 5-FU [48]. These results suggest that the TSER genotyping is essential to select patients capable of responding to a treatment with 5-FU. On the other hand, a large prospective analysis of Niedzwiecki et al. (2016) found that it is useless to assign a specific adjuvant therapy to treat colorectal cancer by measuring the TYMS levels in tumors [49]. According to Marsh et al. (2015), the TSER polymorphism had a variant allele frequency of 0.66 in Peruvian population and 0.65 in Mexican population [50].

### 3.4. Drug-Metabolizing Enzymes

There are more than 30 families of drug-metabolizing enzymes; the genes that encode these proteins are capable of accumulating genetic variants that cause functional changes; therefore, drug metabolism is affected [51]. The antitumor action of the 6-mercaptopurine (6-MP), mainly used to treat leukemia, lies in inhibiting the generation of nucleotides, which are essential for RNA and DNA synthesis. The *S*-methylation of 6-MP, important in the development of inactive metabolites, is catalyzed by thiopurine methyltransferase (*TPMT*) [52,53]. According to Evans et al. (1991), there are patients that have presented hematologic toxicity when treated with 6-MP under the presence of the TPMT*2, TMPT*3A, and TPMT*3C polymorphisms [53]. Each one of these mutant alleles encodes TPMT proteins that are rapidly degradated, generating enzyme deficiency. The frequencies of the *TPMT* alleles differ among ethnic groups [54,55]. For instance, the frequency of *TPMT* mutations was 0.31 (TPMT*3A), 0.07 (TPMT*2), and 0.03 (TPMT*4) in Argentina; 0.00 (TPMT*2), 0.09 (TPMT*3B), and 0.08 (TPMT*3C) in Peru; and 0.00 (TPMT*2), 0.04 (TPMT*3B), and 0.04 (TPMT*3C) in Mexico [50] (Table 1). Consequently, a profile analysis of the *TPMT* deficiency is recommendable to determine a right dose and to avoid toxicity in a treatment with 6-MP on patients with acute lymphatic leukemia [56].

Irinotecan is an antineoplasic drug whose active metabolite is the 7-ethyl-10-hydroxycamptothecin (SN-38), which is capable of inhibiting the topoisomerase-I [57]. Irinotecan is a popular chemotherapy agent since its antitumoral activity is strong. The clinical pharmacogenetics of irinotecan is associated with the presence of SNPs in the UDP-glucuronosyltransferase 1A1 (UGT1A1) enzyme, which is in charge of the glucuronidation of SN-38 to generate the inactive metabolite SN38G [58]. The presence of several repeats TA in the *UGT1A1* gene promoter generates reduced enzyme expression and activity [59,60]. The allele frequencies of UGT1A1*6 reach up to 3.5% in a Caucasian population (Finland), whereas the frequency in Asian populations is higher. In Latin American population, the allele frequencies of UGT1A1*6 was 0.00 in Peru, and 0.13 in Mexico [50] (Table 1).

The cytochrome P450 enzymes are an important family of drug metabolizing enzymes since they catalyze metabolism of more medications than other enzyme families. Debrisoquine hydroxylase (CYP2D6) is the most typical polymorphism of the P450 enzymes worldwide. Subsequently, >30 drugs were found to be substrates for CYP2D6. Polymorphisms of the *CYP1A1* gene are considered as possible breast cancer risk factors since they act as mediators in the tumorigenesis caused by the metabolism of estrogens, metabolic pathway where the enzyme encoded by this gene is involved because it catalyzes several steps in the biosynthesis of steroid hormones, such as estrogen. Its derivative metabolites have an important anti-proliferative and anti-angiogenic activity, whereas other products of the metabolism of estrogen may join the DNA and damage it, suggesting that estrogen and the intermediary products of its metabolism may turn into potential carcinogens [61].

### 3.5. Pharmacogenomics in Clinical Practice

The Canadian Pharmacogenomics Network for Drug Safety (CPNDS), the Royal Dutch Association for the Advancement of Pharmacy (DPWG), and the Clinical Pharmacogenetics Implementation Consortium (CPIC) have created and published precise guidelines for the application of pharmacogenomics in clinical practice [62,63,64]. However, according to a research conducted by Quiñones et al. (2014), there are important barriers to implement the use of pharmacogenomics testing in clinical practice. Some of the barriers that can be found in Latin America are the following: Need for a regional clear guidelines for the use of pharmacogenomics, fragmentation of healthcare systems, insufficient use of electronic records information of patients, lack of knowledge in pharmacogenomics on the part of clinicians, insufficient pharmacogenomic characterization of the target population, insufficient characterization of pharmacogenetic variability in Latin America, lack of health institutions that focus on the development of pharmacogenetic tests, healthcare system do not promote pharmacogenomics use, concerns about test costs, need for implementation of gene/drug pairs, lack of clear information about mutations actually has functional relevance, need for demonstration of clinical validity and utility of pharmacogenomics test, reluctance of clinicians to use genetic biomarkers as markers of clinical response, insufficient definition of the clinical impact of SNPs on specific drugs, and ethical, legal, and social implications [65].

However, overcoming the obstacles previously mentioned with planning will make it possible to get several benefits. Pharmacogenomic tests are capable of improving patient safety; in other words, individuals who are likely to experience dangerous reactions to drugs could be identified, leading to the adjustment of drug doses in a personalized manner. Pharmacogenomics allows for improving the investment in public and private health in the Latin American countries, saving time and resources that doctors and patients need, finding adequate treatments based on “trial and error”. An additional benefit has to do with the improvement of drug dosage used in the different treatment plans against cancer, starting from the genetic chart of each patient rather than their age and weight [31].

Pharmacology of the future intends to conduct individualized pharmacotherapeutic treatment for the manifestation of a disease and the appropriate dose for the therapeutic effect in a given patient, minimizing the risk of adverse reactions. In order to implement successful pharmacogenomics tests in clinical practice at the hospitals in Latin America, it is important to understand the interethnic and intraethnic genetic variability of its populations [60,65].

## 4. Germline Cancer Predisposition in Latin America

Countries in Latin America have undergone significant economic and social changes during the last few decades [66,67]. Declines in reproductive patterns, urbanization and increases in life expectancy are leading to major changes in the population structure and associated increases in the burden of non-communicable diseases, including cancer [66,68].

The GLOBOCAN project has estimated that approximately 14 million cancer cases and 8.2 million cancer deaths were estimated to have occurred worldwide in 2012, with one million cancer cases and more than half a million cancer deaths occurring in Latin America [69]. Projections indicate that by the year 2030, 1.7 million new cases and one million cancer deaths are expected to occur in the region because of ageing and population growth [1,70]. In 2012, GLOBOCAN indicates that the most common cancer diagnoses and causes of cancer-related death in Latin America region were prostate, lung, colorectum, and stomach cancers for males and breast, cervix, colorectum and lung for females [1]. Although cancer incidence in Latin America is, in general, lower than the cancer incidence in more developed regions of the world, mortality is remarkably higher. This may be explained in part by more advanced stages at diagnosis and by poorer access to cancer diagnostic, screening and treatment services [71].

### 4.1. Cancer Incidence and Mortality in Latin America

The incidence rate has increased over the years and the high mortality rate in Latin American populations in contrast with other populations worldwide are warning signs for governments and public and private health institutions to invest in pharmacogenomics research and in the development of effective therapeutic bulls eye to guide medicinal accuracy. The incidence and mortality rates of the most prevalent types of cancer in Latin American populations are detailed below.

**Figure 1:**
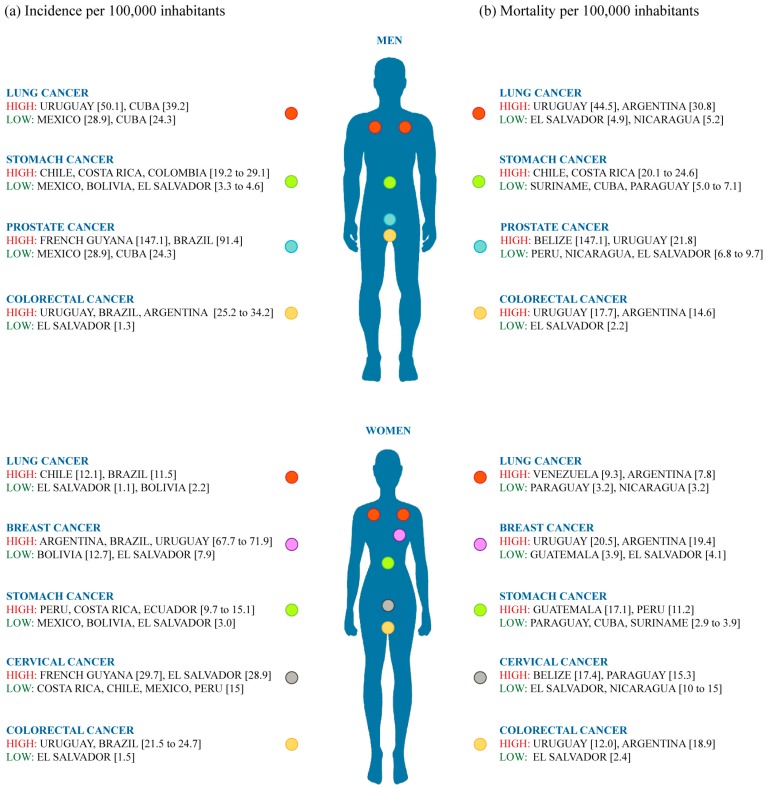
Cancer incidence and mortality in Latin America. (**a**) Cancer incidence per 100,000 inhabitants in men and women; (**b**) Cancer mortality per 100,000 inhabitants in men and women.

Breast cancer was the most frequent cancer diagnosis among females in Latin America, except in Bolivia and El Salvador. The highest age-standardized incidence rates were in Argentina, Brazil, and Uruguay (between 67.7 and 71.9 per 100,000 inhabitants) and the lowest in Bolivia (12.7) and El Salvador (7.9) (Figure 1a). BC was one of the two leading causes of cancer death among females, with the exceptions of Ecuador, Peru, Nicaragua, and Guatemala [72] (Figure 1b). Prostate cancer was the most common malignancy diagnosed among males in Latin America, except in El Salvador and Cuba where it ranked second (after stomach and lung, respectively). During the most recent five-year period (2003–2007), the incidence of prostate cancer varied by six-fold across this region. The highest age-standardized rates were observed in French Guyana (147.1) and Brazil (91.4) and the lowest were in Mexico (28.9) and Cuba (24.3). Prostate cancer was one of the two leading causes of cancer deaths in males in Latin America, except in Chile, Argentina, Colombia and El Salvador where it ranked third. Mortality rates varied by four-fold, with the highest rates seen in Belize (28.9), Uruguay (21.8), and Cuba (24.1) and the lowest in Peru, Nicaragua, and El Salvador (rates between 6.8 and 9.7) [73]. Lung cancer incidence rates ranged from as high as 50.1 among males in Uruguay, to as low as 1.1 among females in El Salvador. In Central America, Cuba had by far the highest incidence of lung cancer for both males and females (39.2 and 18.9, respectively), with lung cancer also ranking in the first place for cancer incidence among males. The South American countries where the highest male incidence rates were observed were Uruguay (50.1), Chile (33.8) and Argentina (30.5). Among females, the pattern was slightly different with the highest incidence rates found in Chile (12.1) and Brazil (11.5). The highest male-to-female incidence ratio was found in Uruguay (5.5) and the lowest in Bolivia (0.6). For mortality, the countries with the highest rates among both males and females in Central America were Cuba (39.0 and 18.1), Belize (15.9 and 6.5) and Mexico (13.2 and 5.4). Among males, lung cancer mortality patterns with the highest rates were Uruguay (44.5), Argentina (30.8) and Chile [39]. In females, countries with the highest lung cancer mortality rates were Venezuela (9.3), Argentina (7.8), Colombia (7.7) and Brazil (7.6) [74]. During the last five years, stomach cancer was one of the five most frequently diagnosed cancers in Argentina, Brazil, Bolivia, Chile, Colombia, Costa Rica, Ecuador, El Salvador, French Guyana and Peru, and one of the five leading causes of cancer death in most Latin American countries (except for females in Argentina, Cuba, and Suriname). In males, the highest incidence rates of stomach cancer were observed in Chile (29.1) followed by Costa Rica, Colombia, Ecuador and Brazil, and Peru (19.2 to 26.5) while the lowest rates were observed in Mexico, Bolivia and El Salvador (3.3 to 4.6). In females, the highest rates were seen in Peru, Costa Rica, Ecuador, Colombia, Chile and Brazil (9.7–15.1) and the lowest rates were in Mexico, Bolivia and El Salvador (3.0). Mortality rates of stomach cancer varied by 5–6-fold. In males, the highest mortality rates were observed in Chile and Costa Rica (20.1–24.6) and the lowest rates were in Suriname, Cuba and Paraguay (5.0–7.1). In females, the highest rates were seen in Guatemala (17.1) followed by Ecuador and Peru (10.5–11.2) and the lowest rates were in Paraguay, Argentina, Cuba and Suriname (2.9–3.9) [75]. Colorectal cancer was among the five most common cancers diagnosed in males and females (except in El Salvador, where it ranked seventh and ninth, respectively) and one of the eight most frequent causes of cancer deaths in Latin America. The highest incidence rates for males and females were observed in Uruguay (34.2 and 24.7, respectively), Brazil (27.7 and 21.5) and in males in Argentina (25.2), whereas the lowest rates were in El Salvador (1.5 and 1.3). Overall, mortality rates were below 10 for both males and females, except in Uruguay (17.7 for males and 12.0 for females), Cuba (10.0 for males and 11.3 for females) and males in Argentina (14.6) [76]. Additionally, cervical cancer was the leading female cancer diagnosis in El Salvador and Bolivia and the second leading female cancer in Mexico, Argentina, Colombia, Ecuador, French Guyana, and Peru. The highest incidence was observed in French Guyana and El Salvador (29.7 and 28.9), while Costa Rica, Chile, Mexico and Cuba showed rates under 15. The highest mortality rates were observed in Belize and Paraguay (17.4 and 15.3, respectively), and in descending order: El Salvador, Nicaragua, Venezuela, Suriname, and Ecuador, with rates between 10 and 15. The lowest mortality was observed in Chile, Uruguay, Brazil and Costa Rica (rates ranging from 6.0 to 7.3) [77].

### 4.2. Genotyping the Latin American Populations with Cancer

It is known that some genetic markers (characteristics of proteins, enzymes, chromosomes, and immunology) are related to different types of cancer. This means that a person with a specific genetic marker is more likely to develop a given illness than another person without the same marker. It should also be consider that in this century most drugs developed for the treatment of cancer are “targeted drugs” forward molecular targets and its effect mainly depends on genetic variants that may be present in the tumor.

Latin America is a region where its populations have different phenotypic characteristics due to the great interracial mixing. Population mixing; consequently, determines an important genetic flow leading to the appearance of complex characteristics influenced by given geographic and environmental factors that allow individuals to get adapted to the region where they live. These evolving changes, most of them subtle, establish a profile that could help to develop pharmacogenomic therapies on populations and individuals in order to take control of diseases, toxicity, and economic investment [35].

Scientists from different research, centers, and hospitals in Latin America have published dozens of articles in SCOPUS database. These articles focus on the population studies of different genes involved in the risk to develop different types of cancer.

Among the most distinguished research, the polymorphisms in the *CYP1A1*, *GSTM1*, *TP53*, *CYP2E1* and *EGFR* genes were significant in lung cancer in Latin Americans (*p* < 0.05) [78,79,80,81], as well as in Caucasians and Asians (Table 2) [82,83,84,85,86,87,88]. Mutations in the *BRCA1/2* and *MTHFR* genes in Latin Americans were associated with breast and ovarian cancer [89,90,91,92,93,94,95,96,97,98,99,100,101,102,103], also observed in Asians and Caucasians [104,105,106,107]. Mutations in the *CYP1A1*, *GSTM1*, *MTHFR*, *SRD5A2*, *AR*, and *GSTA1* genes were relevant in prostate cancer in Latin Americans [108,109,110,111,112], also determined in Caucasians and Asians [113,114,115,116,117,118,119,120,121]. The *IL-1*, *TP53*, *WNT*, 8q24 region and *IL-8* genes were consistent with gastric cancer in Latin Americans [122,123,124,125,126], as well as in some Asians and Caucasians [127,128,129,130]. The *CDKN2A* and *MC1R* genes were related with myeloma in Latin Americans [131], as well as in Caucasians [132,133]. Polymorphisms in the *GPX1*, *GST* and *NQO1* genes were suitable with bladder cancer [134,135], as well as in Caucasians and Asians [136,137,138,139]. Mutations in the *CCND1* and *TP53* genes were significant in colorectal cancer [140,141], also observed in Caucasians and Asians [142,143,144,145]. Gene alterations in *GSTM1* were associated with larynx cancer en Latin Americans [146], as well as in Asians [147,148]. Mutations in the *CYP1A1* and *GSTM1* genes were relevant in oral cancer in Latin Americans [149], also detected in Asians [150,151,152]. The *hRAD54*, *hMSH2*, *ABCB1*, *ABCC5*, *COL18A1*, and *SLC19A1* genes were consistent with lymphoma and leukemia in Latin Americans [153,154,155,156], as well as some Caucasians and Asians [157,158,159,160]. The *RB1* gene was related with retinoblastoma in Latin Americans [161,162], and the *TNFα* and *TP53* genes with cervical cancer [163,164,165,166,167,168].

**Figure 2:**
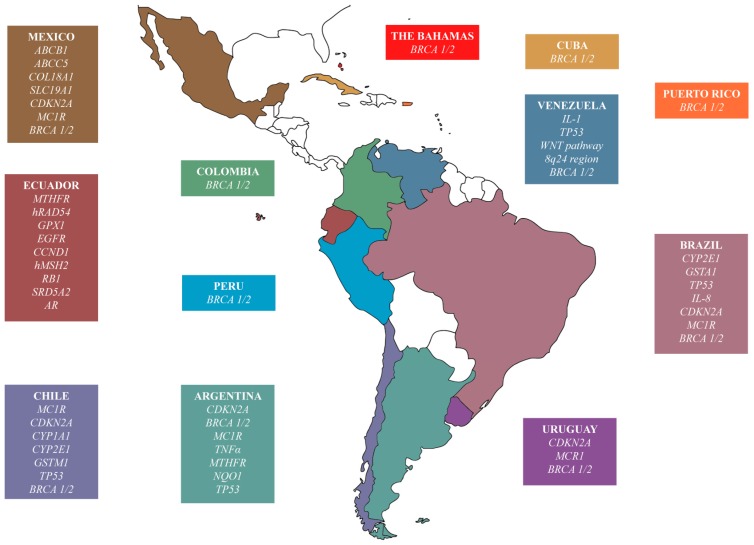
Cancer associated genes studied by country in Latin America.

All these researches have determined the association of different genetic variants with the highest risk to develop different types of cancer in the Latin American populations. These results allow the understanding of the genome both at population and individual level in the best possible manner turning this a relevant step for the development (Table 2) (Figure 2).

## 5. Conclusions

This is a transformative time for cancer therapy. The feasibility of establishing the detailed molecular portraits of individual cancers, even at the point of care, is no longer the primary obstacle to progress. Similarly, new highly potent and selective purpose-built inhibitors are being developed at a time when our understanding of actionable mutations in cancer genomes is improving steadily, resulting in continuous erosion of the market share of what has been called the undruggable genome [169]. Over the last decade, pieces of research in silico and experiments to detect somatic and germline alterations across tumor genomes have been conducted. The last progress in DNA sequencing technologies and high inter-tumor heterogeneity has generated results that are difficult to understand to identify the genes and pathways involved in tumorigenic process. Consequently, new customized medicine strategies will make it possible to improve targeted therapies against the drivers of cancer and this way, to improve patients’ quality of life [170].

The implementation of pharmacogenomics in health policies conveys many benefits to patients who suffer from different types of cancer. There are several projects in charge of analyzing genomes, finding DNA elements, genes and genetic variations that affect health and disease, such as The Cancer Genome Atlas Network, the 1000 Genomes Project, the International HapMap Project, and the ENCODE Project [34,171,172,173]. Nevertheless, most of the samples analyzed in these projects belong to European, Asian, North American, and African populations, being the genetic participation and characterization in Latin American populations very limited [34,171,172,173]. The HapMap project is one of the first and largest efforts to characterize germline variant frequencies across racial populations and did not have any Latin American or South American populations (only a Mexican population in the United States) [172]. The 1000 Genomes Project (phase 3) has analyzed patients from Mexico, Colombia, Peru, and Puerto Rico [34].

The capacity to map and understand human genome has been a great success in cancer research. Starting from the analysis of the somatic and germline genomes, it is possible to establish an efficient anticancer therapy. The germline genome is involved in determining drug toxicity and exposure. The somatic genome may predict tumor behavior if treated (efficacy prediction) or untreated (prognosis) [5]. The pioneering example of molecularly driven cancer medicine was the development and use of the kinase inhibitor imatinib for the treatment of chronic myelogenous leukemias that harbor the *BCR*-*ABL1* balanced chromosomal translocation [169]. Similarly, the advent of HER2-targeted therapies for the treatment of women with newly diagnosed metastatic HER2-positive breast cancer has radically changed the outcome of what was until recently the most lethal form of breast cancer [169].

Worldwide, there are clear guidelines for the use of pharmacogenomics published by the Canadian Pharmacogenomics Network for Drug Safety (CPNDS), the Royal Dutch Association for the Advancement of Pharmacy (DPWG), and the Clinical Pharmacogenetics Implementation Consortium (CPIC) [62,63,64]. For instance, simvastatin is effective in patients with the *SCLO1B1* T37041C polymorphism; thioguanine is effective in individuals with the TPMT*2, *3A, *3C and *4A polymorphisms; atazanavir and irinotecan are effective in patients with the UGT1A1*28 polymorphism; and amitriptyline, carvedidol, doxepin, haloperidol, olanzapine, paroxetine and venlafaxine are effective in individuals with the CYP2D6 polymorphism [62,63,64]. Regarding allele frequencies for these clinically relevant germline polymorphisms, Latin American countries (Colombia, Mexico, Peru, Puerto Rico, and Argentina) have similar frequencies in their alleles in contrast with Caucasian countries (Spain, England, Scotland, Finland, Italy and United States), and Asian countries (China, Bangladesh, Japan and Vietnam) [34,172].

The genotyping studies performed in 12 Latin American countries revealed 26 significant cancer driver genes implicated in 12 cancer types, such as lung, breast, ovarian or colorectal cancer. Many of these genes were significant in Latin America countries but not Caucasian or Asian populations. For instance, *CYP2E1* and *GSTM1* were significant in Brazil and Chile, respectively, but not in Caucasians or Asians. *MTHFR* was significant in Ecuador, but not in Caucasians. *IL-1* was significant in Venezuela, but not in Asians (please see Table 2 for details) [80,88,103,106].

The progress in pharmacogenomics worldwide and the implementation of knowledge and techniques in clinical practice will make it possible to overcome the barriers found in Latin America. For instance, it is possible to develop regional pharmacogenomics guidelines to create health institutions in charge of developing pharmacogenetic tests, to improve health care systems by promoting the progress on pharmacogenomics, to invest in pharmacogenomics variability in Latin American populations for implementation of gene/drug pairs, to implement electronic records information of patients, and to improve economic, ethical, legal and social implications. Likewise, these goals should include ethnic comparison of pharmacogenomic profiles, gene expression, and regulation; the impact of polymorphism on phenotype; metabolic profiles of patients with a given drug; and relevant environmental factors that influence drug response. Therefore, the main idea is to accomplish the five “R” for drug therapy: “the Right dose of the Right drug for the Right indication in the Right patient at the Right time” [65].

## Figures and Tables

**Table 1:** Allele frequencies for clinically relevant germline polymorphisms in populations worldwide according to the 1000 Genomes Project (Phase 3) and HapMap Project.

Gene	Polymorphism	Human Populations		
		Latin America	Caucasian	Asian
*SLCO1B1*	T37041C (rs4149056)	Colombia: 0.08 (C) **;	Spain: 0.12 (C);	Han Chinese: 0.14 (C);
		Mexico: 0.08 (C);	British: 0.14 (C);	Bangladesh: 0.05 (C);
		Peru: 0.14 (C);	Finland: 0.18 (C);	Japan: 0.12 (C);
		Puerto Rico: 0.12 (C)	Italy: 0.21 (C)	Vietnam: 0.10 (C)
*TYMS*	TSER*2/*3/*4 (rs34743033)	Self-described Hispanic: 0.41 (*2) ***;	Self-described Caucasian: 0.45 (*2);	Pacific Rim: 0.11 (*2); 0.89 (*3)
		Self-described Hispanic: 0.59 (*3);		
		Peru: 0.66 (*4);	Self-described Caucasian: 0.55 (*3)	
		Mexico: 0.65 (*4)		
*TPMT*	TPMT*2 (rs1800462)	Colombia: 0.01 (G);	Spain: 0.01 (G);	Han Chinese: 0.00 (G);
		Mexico: 0.00 (G);	British: 0.01 (G);	Bangladesh: 0.00 (G);
		Peru: 0.01 (G);	Finland: 0.00 (G);	Japan: 0.00 (G);
		Puerto Rico: 0.01 (G)	Italy: 0.99 (C), 0.01 (G)	Vietnam: 0.00 (G)
	TMPT*3A (rs1800460)	Colombia: 0.01 (T);	Spain: 0.04 (T);	Han Chinese: 0.00 (T);
		Mexico: 0.04 (T);	British: 0.03 (T);	Bangladesh: 0.02 (T);
		Peru: 0.06 (T);	Finland: 0.03 (T);	Japan: 0.00 (T);
		Puerto Rico: 0.05 (T)	Italy: 0.01 (T)	Vietnam: 0.00 (T)
	TPMT*3C (rs1142345)	Colombia: 0.02 (C);	Spain: 0.04 (C);	Han Chinese: 0.01 (C);
		Mexico: 0.05 (C);	British: 0.03 (C);	Bangladesh: 0.03 (C);
		Peru: 0.06 (C);	Finland: 0.03 (C);	Japan: 0.02 (C);
		Puerto Rico: 0.10 (C)	Italy: 0.01 (C)	Vietnam: 0.03 (C)
	TPMT*4A (rs1800584)	Argentina: 0.03 (A); Mexico: 0.04 (A)	Utah residents with Northern and Western European ancestry: 0.01 (A); Italy: 0.01 (A)	-
*UGT1A1*	UGT1A1*6 (rs4148323)	Colombia: 0.03 (A);	Spain: 0.00 (A);	Han Chinese: 0.23 (A);
		Mexico: 0.02 (A);	British: 0.00 (A);	Bangladesh: 0.03 (A);
		Peru: 0.00 (A);	Finland: 0.04 (A);	Japan: 0.13 (A);
		Puerto Rico: 0.00 (A)	Italy: 0.00 (A)	Vietnam: 0.07 (A)

** Frequency of minor allele, *** Number of repeats per mutation.

**Table 2:** Genotyping studies related with several types of cancers in human populations worldwide.

Disease	Gene	Human Populations			Reference
		Latin American (Country)	Caucasian *	Asian *	
Lung cancer	*CYP1A1*	√ (Chile)	√	√	[78,83,84]
	*GSTM1*	√ (Chile)	√	√	[78,83,85]
	*TP53*	√ (Chile)	√	√	[79,86,87]
	*CYP2E1*	√ (Brazil)	Ø	Ø	[80,88]
	*EGFR*	√ (Ecuador)	√	√	[81,82]
Breast cancer/ovarian cancer	*BRCA1*	√ (Chile, Venezuela, Colombia, Mexico, Argentina, Brazil, Puerto Rico, Uruguay, Peru, The Bahamas)	√	√	[89,90,91,92,93,94,95,96,97,98,99,100,101,102,104,105]
	*BRCA2*	√ (Chile, Venezuela, Colombia, Mexico, Argentina, Brazil, Puerto Rico, Uruguay, Peru, The Bahamas)	√	√	[89,90,91,92,93,94,95,96,97,98,99,100,101,102,104,105]
	*MTHFR*	√ (Ecuador)	Ø	√	[103,106,107]
Prostate cancer	*CYP1A1*	√ (Chile)	Ø	√	[108,113]
	*GSTM1*	√ (Chile)	Ø	Ø	[108,114]
	*MTHFR*	√ (Ecuador)	√	√	[109,115]
	*SRD5A2*	√ (Ecuador)	√	Ø	[110,116,117]
	*AR*	√ (Ecuador)	√	√	[111,118,119]
	*GSTA1*	√ (Brazil)	√	√	[96,120,121]
Gastric cancer	*IL-1*	√ (Venezuela)	√	Ø	[122,127]
	*TP53*	√ (Venezuela)	√	√	[123,128]
	*WNT*	√ (Venezuela)	-	-	[124]
	*8q24*	√ (Venezuela)	√	√	[125,129]
	*IL-8*	√ (Brazil)	Ø	√	[126,130]
Melanoma	*CDKN2A*	√ (Chile, Mexico, Argentina, Brazil, Uruguay)	√	-	[131,132]
	*MC1R*	√ (Chile, Mexico, Argentina, Brazil, Uruguay)	√	-	[131,133]
Bladder cancer	*GPX1*	√ (Ecuador)	√	√	[134,136,137]
	*GST*	√ (Argentina)	√	√	[135,138]
	*NQO1*	√ (Argentina)	Ø	√	[135,139]
Colorectal cancer	*CCND1*	√ (Ecuador)	√	√	[140,142,143]
	*TP53*	√ (Argentina)	Ø	√	[141,144,145]
Larynx cancer	*GSTM1*	√ (Chile)	Ø	√	[146,147,148]
Oral cancer	*CYP1A1*	√ (Chile)	Ø	√	[141,142,143]
	*GSTM1*	√ (Chile)	Ø	√	[149,150,152]
Lymphoma/leukemia	*hRAD54*	√ (Ecuador)	-	-	[153]
	*hMSH2*	√ (Ecuador)	√	Ø	[154,157,158]
	*ABCB1/5*	√ (Mexico)	√	√	[155,156,159,160]
	*COL18A1*	√ (Mexico)	-	-	[156]
	*SLC19A1*	√ (Mexico)	-	-	[156]
Retinoblastoma	*RB1*	√ (Ecuador)	-	√	[161,162]
Cervical cancer	*TNFα*	√ (Argentina)	√	√	[163,165,166]
	*TP53*	√ (Brazil)	√	√	[164,167,168]

√ Significant, Ø Non-significant, - Not found, * Meta-analysis studies.
